# The Pharmacokinetics, Bioavailability, and Excretion Studies of α-Cyperone in Rats by UHPLC-QQQ-MS/MS

**DOI:** 10.3390/molecules30193899

**Published:** 2025-09-26

**Authors:** Ye Shang, Yameng Zhu, Kaili Zhang, Zijing Zhang, Huining Geng, Xueyu Liu, Wenwen Li, Lu Chen, Caixia Li, Yang Liu, Huizi Ouyang, Jun He

**Affiliations:** State Key Laboratory of Chinese Medicine Modernization, Tianjin University of Traditional Chinese Medicine, Tianjin 301617, China; sytjutcm@126.com (Y.S.); yameng354@163.com (Y.Z.); zkl101033@163.com (K.Z.); zzj010712@163.com (Z.Z.); genghuining0304@163.com (H.G.); liuxy100805@163.com (X.L.); lww1981896@163.com (W.L.); cl15515006158@163.com (L.C.); lcxtcm@126.com (C.L.); yeeliuyang@163.com (Y.L.); huihui851025@163.com (H.O.)

**Keywords:** α-cyperone, pharmacokinetic, bioavailability, excretion, UHPLC-QQQ-MS/MS

## Abstract

*α*-Cyperone (C_15_H_22_O), a critical bioactive sesquiterpene, serves as a representative chemical compound of *Cyperi Rhizoma*—a classical functional food. To investigate the pharmacokinetic characteristics of α-cyperone, a quantified method was developed in plasma, bile, urine, and feces by ultra-high-performance liquid chromatography tandem triple quadrupole mass spectrometry (UHPLC-QQQ-MS/MS). After being validated, the developed method was applied in a plasma pharmacokinetic study as well as biliary, urinary, and fecal excretion kinetics studies. It revealed poor absolute bioavailability (F = 1.36%) and rare excretion (total cumulative excretion = 0.022%) of α-cyperone, which suggested extensive first-pass metabolism. This study provided crucial insight into explaining the in vivo process and promoting the further development of α-cyperone.

## 1. Introduction

*Cyperi Rhizoma* (CR) is the dried rhizome and root of *Cyperus rotundus* L., with a homology of medicine and food [1]. As a kind of functional food, the safety and efficiency of CR benefits from its unique phytochemical composition, such as volatile oil, flavonoids, and phenolic acid [2,3]. Notably, the volatile oil is the major contributor to the biological function and aroma of CR, which has prompted the widespread use of CR as a traditional medicine and spice [4]. Among the volatile oils, *α*-cyperone (C_15_H_22_O) is one of the most characteristic compounds. It possesses significant pharmacological properties for the treatment of Parkinson’s disease [5], osteoarthritis [6], diabetic nephropathy [7], etc. Furthermore, *α*-cyperone has played an indispensable role in combination drugs against gynecological diseases.

The exertion of biological function depends on a clear dynamic process in vivo of active ingredients [8]. Pharmacokinetics study has been devoted to describing a clear profile of absorption, distribution, metabolism, and excretion (ADME) in vivo [9,10]. The pharmacokinetic characteristics of *α*-cyperone have been reported in some extracts of phytomedicine [11,12,13]. It could be found in a reported study that α-cyperone possessed weak plasma exposure, even if a high oral dosage was administered. In order to clarify the process of *α*-cyperone in vivo, it is essential to independently investigate the compound rather than the extract in order to avoid drug–drug interactions caused by the complex matrix of the medicinal plant [14,15]. Therefore, an essential piece of information can be provided through a bioavailability study of *α*-cyperone.

*α*-Cyperone is a kind of eudesmane-type sesquiterpene. Its featured chemical structure manifests attractive pharmacological advantages, which indicate worse water solubility (LogP = 4.22). The corresponding effect of absorbing drug molecules would be unsatisfactory, which would promote direct excretion [16,17,18,19]. Alternatively, extensive first-pass metabolism might occur prior to systemic circulation. Most of the *α*-cyperone would be directly consumed by the metabolic enzyme [20,21]. Therefore, a satisfactory explanation about the weak plasma exposure can be obtained via investigating the excretion characteristics of α-cyperone.

In this study, an ultra-performance liquid chromatography tandem triple quadrupole mass spectrometry (UHPLC-QQQ-MS/MS) method was developed to quantify α-cyperone in rat plasma, bile, urine, and feces for application in pharmacokinetics, bioavailability, and excretion studies for the first time. It was able to further illustrate the in vivo process and promote the application prospect of *α*-cyperone in functional food.

## 2. Results and Discussions

### 2.1. Optimization of Sample Preparation

The type of extraction solvent is commonly regarded as a critical parameter in biological sample preparation. In this work, the kind of extraction solvent (methanol, acetonitrile, and ethyl acetate) and volume of formic acid (0 and 5 μL) were utilized to investigate the optimal extraction program. For the extraction solvent of liquid sample (plasma, urine, and bile), methanol and acetonitrile were utilized for the protein precipitation; meanwhile, ethyl acetate was employed for liquid–liquid extraction. It is worth noting that neither methanol nor acetonitrile was suitable for the urine sample; the extraction recovery of methanol was only 22.7 ± 1.7% and the matrix effect of acetonitrile was 60.5 ± 1.2%. Comparatively, the liquid sample preparation method was satisfactory when the ethyl acetate mixed with 5 μL of formic acid was used as a solvent to extract α-cyperone. For the feces preparation, formic acid was able to cause serious suppression of ionization, and the matrix effect was no more than 37.8%. As a result, acetonitrile was more suitable for feces.

### 2.2. Optimization of QQQ-MS/MS

**Figure 1:**
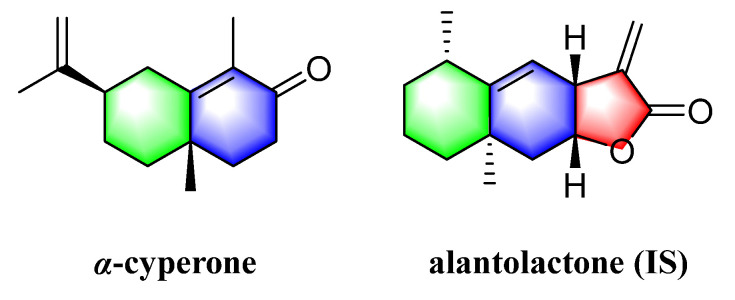
The chemical structure of α-cyperone and alantolactone.

**Figure 2:**
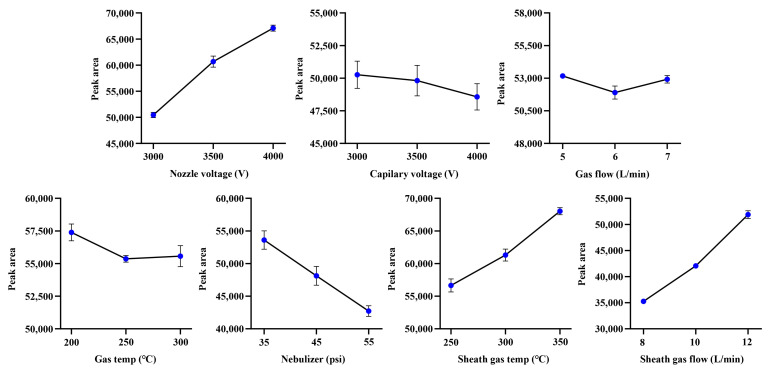
The optimization of ion source parameters (*n* = 3, mean ± SD).

In order to maximize the sensitivity of the developed method for *α*-cyperone as much as possible (Figure 1), a single factor experiment with seven factors and three levels was performed. As the optimization results, sheath gas temp (250–350 °C), sheath gas flow (8–12 L/min), and nozzle voltage (3.0–4.0 kV) could enhance the response of α-cyperone in a level-dependent manner. Conversely, the optimal sensitivity of α-cyperone for capillary voltage (3.0–4.0 kV), nebulizer (35–55 psi), gas flow (5–7 L/min), and gas temp (200–300 °C) was achieved at the lowest level (Figure 2). As a result, the ion source parameters were established as follows: gas temp, 200 °C; gas flow, 5 L/min; nebulizer, 35 psi; sheath gas temp, 350 °C; sheath gas flow, 12 L/min; capillary voltage, 3.0 kV; and nozzle voltage, 4.0 kV.

### 2.3. Method Validation of the Constructed UHPLC-QQQ-MS/MS

**Figure 3:**
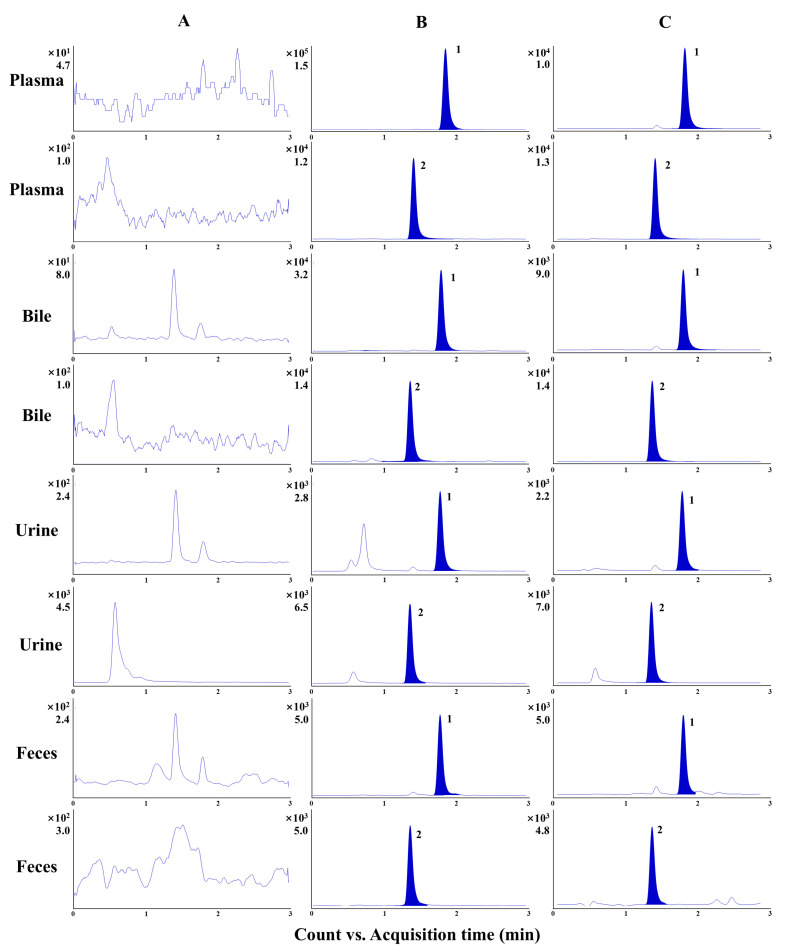
The representative MRM chromatograms of plasma, bile, urine, and feces from a blank sample (**A**), a sample after administration at the earliest period (**B**), and a QC sample at a low concentration level (**C**). The numbers 1 and 2 represent α-cyperone and alantolactone, respectively.

The representative MRM chromatograms were utilized to investigate specificity. No significant interference was observed at the same retention time of α-cyperone and IS (Figure 3). According to the assessment of linearity, α-cyperone could be quantified within a wide range of concentration, followed by a satisfactory correlation coefficient (no less than 0.9996). The LLOQ was in the range of 0.15–0.45 ng/mL or ng/mg of feces, which illustrates that sensitive determination could be performed by the developed method (Table 1). The intra- and inter-day precisions were both within an acceptable range (from 0.4% to 12.4%), with an accuracy of no more than ± 14.5%. In plasma, bile, and urine, the extraction recovery and matrix effect of α-cyperone were within the ranges of 85.4–114.6% and 86.3–99.0%, respectively (Table S1). It is worth noting that the α-cyperone in feces possessed enough extraction recovery, while the matrix effect was 72.9–83.7% at three concentration levels. Nevertheless, the corresponding LLOQ was able to reach 0.45 ng/mg, which illustrates that the slight interference from the matrix was not enough to impair the sensitive determination. The stability investigation showed that α-cyperone in biological samples of rats was stable under different conditions (an RE of no more than ± 9.7% with an RSD of no more than 14.0%) (Table S2).

### 2.4. Plasma Pharmacokinetic Study

**Figure 4:**
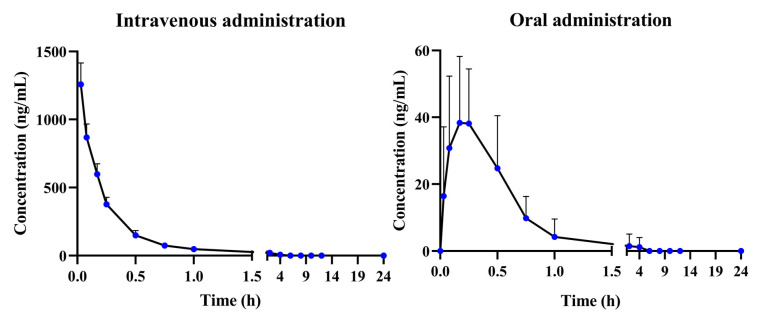
The plasma concentration–time profiles of α-cyperone after intravenous administration (4 mg/kg) and oral administration (20 mg/kg) (*n* = 6, mean ± SD).

The developed and validated UHPLC-QQQ-MS/MS method was employed for the determination of α-cyperone in rat plasma after intravenous and oral administration (Figure 4). After oral administration, the α-cyperone was rapidly absorbed into plasma (T_max_ = 0.20 ± 0.16 h) and completely consumed within 6 h. The half-life time (T_1/2_) was 0.14 ± 0.05 h, which illustrates that the elimination of *α*-cyperone was faster than the absorption. In addition, the short mean residence time (MRT) of α-cyperone (0.47 ± 0.22 h) also suggested rapid elimination of α-cyperone in rat plasma. At an oral dosage of 20 mg/kg, the C_max_ and AUC_(0→t)_ were 51.19 ± 16.41 ng/mL and 25.89 ± 14.01 μg/L × h, respectively, which indicated low plasma exposure of α-cyperone in vivo (Table 2).

At an intravenous dosage of 4 mg/kg, the AUC_(0→t)_ of α-cyperone was 380.62 ± 50.73 μg/L × h. Subsequently, the absolute bioavailability was calculated as 1.36%, which suggested poor absorption in the form of a prototype or rapid metabolism from the prototype to metabolites.

The clinical indication of the drug was designed according to the dynamic characteristics in vivo. It could be found from a plasma pharmacokinetic study that α-cyperone possessed weak exposure accompanied by a short residence time. For most chronic diseases, the plasma concentration required for effectiveness is generally expected to be maintained for as long as possible. As for the reported study, the significant therapeutic efficacy of *α*-cyperone was clarified in chronic kidney disease [22] and diabetic nephropathy [7], which were investigated using an oral dosage of 5–50 mg/kg. It could be inferred that *α*-cyperone could be easily absorbed and utilized in the form of metabolites before excretion. Therefore, it was necessary to explore the behavior of *α*-cyperone in excretion.

### 2.5. Excretion Kinetics Study

**Figure 5:**
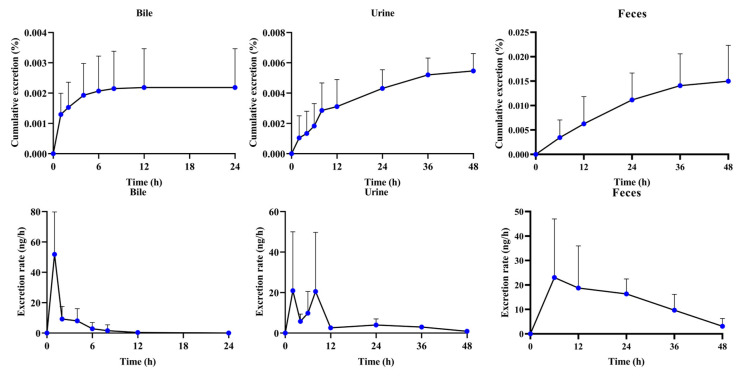
The excretion process after oral administration of α-cyperone (20 mg/kg) in rat bile, urine, and feces (*n* = 6, mean ± SD).

The established UHPLC-QQQ-MS/MS method was used to implement biliary, urinary, and fecal excretion kinetics studies to explore the reason for poor bioavailability (Figure 5 and Table 3). It is worth noting that α-cyperone could not be detected in bile, urine, and feces after intravenous administration at a dosage of 4 mg/kg, even with higher plasma exposure. On the contrary, α-cyperone could be detected in the bile, urine, and feces at an oral dosage of 20 mg/kg. The total cumulative excretion was nearly 0.022%. The biliary, urinary, and fecal cumulative excretions accounted for 0.002%, 0.005%, and 0.015% of the dose, respectively. This not only implied that the conversion of α-cyperone in systemic circulation was too fast to be excreted in the form of prototype, but it also indicated that fecal excretion was the major elimination route of α-cyperone.

The quickest biliary, urinary, and fecal elimination rates were 51.8 ng/h (0–1 h after administration), 20.9 ng/h (0–2 h after administration), and 23.0 ng/h (0–6 h after administration), respectively (Figure 5). This suggested that the quickest eliminations all concentrated on the initial stage after administration in the major excretion routes. The excretion T_1/2_ could assist in exploring the indication of α-cyperone. The α-cyperone had a long elimination period due to the elimination T_1/2_ of bile, urine, and feces being 2.10 ± 0.72 h, 2.10 ± 0.72 h, and 18.84 ± 9.15 h, respectively. This was able to reflect the slow excretion of α-cyperone. Perhaps another possibility is that the metabolites of α-cyperone had a long MRT. Few of them could be transformed back to prototypes and excreted outside the body. However, no matter how it was excreted, the total cumulative excretion of α-cyperone was very small. Therefore, it was valuable for the further development of α-cyperone to focus on the in vivo process of its metabolites.

As demonstrated in numerous cellular studies, α-cyperone was able to significantly alleviate inflammatory damage [23], oxidative stress [24], and the proliferation of tumors [25]. However, conventional in vitro activity evaluation systems failed to recapitulate critical pharmacokinetic processes such as gastrointestinal absorption and hepatic metabolism prior to distribution at lesions. Consequently, the actual bioactive form (prototype or metabolites) responsible for in vivo efficacy remains to be investigated. Several efforts have been made to improve dosage forms to enhance oral bioavailability and bioactivity, such as green nanoparticles [26], *β*-cyclodextrin inclusion complexes [11], etc. Nevertheless, it was imperative to conduct a systematic investigation to describe the metabolic process of α-cyperone. Future studies should prioritize the isolation and characterization of the major metabolites, which is helpful to enable comparative bioactivity assessment between the parent compound and its metabolic derivatives.

## 3. Materials and Methods

### 3.1. Regents and Chemicals

Both LC-grade methanol and acetonitrile were bought from Fisher Scientific (Waltham, MA, USA). LC-MS-grade formic acid was obtained from Anaqua Chemicals Supply (Wilmington, DE, USA). Ethyl acetate was provided by Concord Technology Co., Ltd. (Tianjin, China). The ultra-pure water was produced from a Milli-Q water purification system (Billerica, MA, USA). The reference substances of α-cyperone (CAS 473-08-5) and alantolactone (CAS 546-43-0) were purchased from Chengdu Desite Bio-Technology Co., Ltd. (Chengdu, Sichuan, China).

### 3.2. UHPLC-QQQ-MS/MS Condition

The separation of α-cyperone and alantolactone (internal standard, IS) was performed with an Agilent 1290 II infinity UHPLC system (Santa Clara, CA, USA) on a Waters Cortex C18 column (2.1 × 50 mm, 1.7 μm) at 35 °C. The mobile phase was composed of 0.1% formic acid aqueous solution (*v*/*v*) and acetonitrile. The separation procedure was operated through isocratic elution in 65% acetonitrile with a flow rate of 0.3 mL/min for 3 min. The injection volume was set at 2 μL.

An Agilent 6470 QQQ-MS/MS system (Santa Clara, CA, USA) equipped with an air-jet stream electron spray ionization (AJS ESI) ion source was utilized for the determination of target compounds. The positive multiple reaction monitoring (MRM) was selected to achieve high-specificity data acquisition (Table 4). The parameters of the ion source were set as follows: drying gas, 200 °C; gas flow, 5 L/min; nebulizer, 35 psi; sheath gas temp, 350 °C; sheath gas flow, 12 L/min; capillary voltage, 3.0 kV; and nozzle voltage, 4.0 kV.

### 3.3. Biosample Collection

The male Sprague Dawley rats (200 ± 10 g) were bought from Beijing Huafukang Bio-Technology Co., Ltd. (Beijing, China). All of the rats were housed in 40–60% humidity and 23–27 °C and subjected to a 12 h dark–light cycle at the animal center of Traditional Chinese Medicine of Tianjin University. A dosage of 20 mg/kg and 4 mg/kg was administered to rats via oral and intravenous methods, respectively. Free access to water and food was given to animals until 12 h before the experiment. All of the animal studies were performed under the supervision of the Laboratory Animal Ethics Committee of Tianjin University of Traditional Chinese Medicine (TCM-LAEC2024063s1600).

A 220 μL blood sample was separately collected from the ophthalmic venous plexus pre-dose and 0.03, 0.08, 0.17, 0.25, 0.5, 0.75, 1, 2, 4, 6, 8, 10, 12, and 24 h post-dose. All of the blood samples were centrifuged for 10 min at 7000 rpm. The obtained plasma layer was stored at −80 °C before use.

The rats were anesthetized with urethane at a dosage of 1.5 g/kg. Then, intubation surgery was performed to obtain bile continuously. The bile was collected pre-dose and 0–1 h, 1–2 h, 2–4 h, 4–6 h, 6–8 h, 8–12 h, and 12–24 h post-dose. All of the biliary samples were stored at −80 °C before use.

After administration of α-cyperone, the rats were kept in metabolic cages. Subsequently, the urine was collected pre-dose and 0–2 h, 2–4 h, 4–6 h, 6–8 h, 8–12 h, and 12–24 h post-dose. Then, the urine was centrifuged for 10 min at 14,000 rpm. The supernatant was stored at −80 °C before use. Meanwhile, feces were obtained pre-dose and 0–6 h, 6–12 h, and 12–24 h post-dose. The feces were dried and stored in a desiccator before use.

### 3.4. Sample Preparation

Liquid–liquid microextraction was employed for the preparation of plasma, bile, and urine samples. A total of 10 μL of methanol, 10 μL of IS (1 μg/mL), 5 μL of formic acid, and 1 mL of ethyl acetate were added to 100 μL of plasma (100 μL of urine or 100 μL of bile). The mixture was centrifuged for 10 min at 14,000 rpm after vortexing for 3 min. The ethyl acetate layer was transformed in a centrifugal evaporator and dried at 50 °C. The residue was redissolved with 100 μL of methanol and centrifuged for 10 min at 14,000 rpm. Subsequently, the supernatant was used for the UHPLC-QQQ-MS/MS analysis.

For the fecal excretion analysis, a weight of 25 mg of feces was extracted with 10 μL of methanol, 10 μL of IS (1 μg/mL), and 1 mL of acetonitrile by vortexing for 3 min. The supernatant was transformed in a centrifugal evaporator after centrifuging for 10 min at 14,000 rpm. The residue was redissolved with 100 μL of methanol and centrifuged for 10 min at 14,000 rpm. Subsequently, the supernatant was used for the UHPLC-QQQ-MS/MS analysis.

### 3.5. Preparation of Standard and Quality Control Samples

The stock solution of analytes was prepared with methanol to a concentration of 1 mg/mL. The working solutions of α-cyperone were obtained by stepwise diluting the stock solution to desired concentrations with ratios of 2, 2.5, 2, 2.5, 2, 2.5, 2, and 2.5. For the preparation of a calibration sample, 10 μL of working solution and 10 μL of IS (1 μg/mL) were spiked into 100 μL of blank plasma (100 μL of urine, 100 μL of bile, or 25 mg of feces) and extracted in the same manner as the sample preparation. The quality control (QC) sample was prepared in the same manner as the calibration sample and set with three concentration levels—low, medium, and high—for the method validation.

### 3.6. Method Validation

In this work, specificity, sensitivity, linearity, precision, extraction recovery, matrix effect, and stability were utilized to validate the developed method according to the Guidance for industry of Bioanalytical Method Validation and Study Sample Analysis from the Food and Drug Administration (FDA) of the USA. The specificity was assessed by comparing the chromatogram among the blank sample, the QC sample, and the sample after administration of α-cyperone. The lower limit of quantification (LLOQ) was used to represent sensitivity. The LLOQ was calculated by the concentration when the signal-to-noise (S/N) was 10. The calibration curve was constructed using a relative peak area and relative concentration of the ratio of analyte against IS. Correlation coefficient (r) was used for the linearity evaluation. The relative standard deviation (RSD) and relative error (RE) were calculated for the representation of intra-/inter-day precision and accuracy, which were determined using six replicates of QC samples on the same day or across three consecutive days, respectively. The extraction recovery was investigated by comparing the peak area of the analyte in the QC sample with the post-treatment spiked sample at the same concentration. Similarly, the matrix effect was evaluated by comparing the post-treatment spiked sample with the standard solution. The stability was assessed by the RE and RSD of α-cyperone in QC samples stored at room temperature for 4 h, in an autosampler for 12 h, at –80 °C refrigerator for 7 days, and undergoing three cycles of freeze and thaw.

### 3.7. Data Analysis

The Drug and Statistics 3.0 (Medical College of Wan-nan, China) software was used to characterize pharmacokinetic parameters of α-cyperone with a non-compartmental statistical model, such as maximum plasma concentration (C_max_), time to reach C_max_ (T_max_), and half-life time (T_1/2_). The obtained area under the curve (AUC) was used to calculate the absolute bioavailability (F) with the following formula:

(1) $$F=\frac{{\mathrm{AUC}}_{\left(\mathrm{oral}\right)}\times {\mathrm{Dosage}}_{\left(\mathrm{intravenous}\right)}}{{\mathrm{AUC}}_{\left(\mathrm{intravenous}\right)}\times {\mathrm{Dosage}}_{\left(\mathrm{oral}\right)}}\times 100\%$$

## 4. Conclusions

Herein, a rapid, sensitive, and reliable UHPLC-QQQ-MS/MS method was developed and validated to quantify α-cyperone in plasma, bile, urine, and feces of rats. The plasma pharmacokinetic study suggested the quick absorption (T_max_ = 0.20 ± 0.16 h), rapid metabolism (T_1/2_ = 0.14 ± 0.05 h), and poor bioavailability (F = 1.36%) of α-cyperone. Additionally, the biliary, urinary, and fecal elimination studies proved rare excretion of α-cyperone (total cumulative excretion was 0.022% of the dose). Considering the in vivo process, it could be deduced that α-cyperone was rapidly metabolized after administration and transferred into systemic circulation. This study is meaningful for the pharmacological study and further development of α-cyperone.

## Figures and Tables

**Table 1:** The linear range, regression equation, correlation coefficient (r), and LLOQ of α-cyperone in plasma, bile, urine, and feces.

Sample	Liner Range (ng/mL or ng/mg)	Regression Equation	r	LLOQ (ng/mL or ng/mg)
plasma	2.4–1500	y = 1.9160x + 0.2433	0.9999	0.15
bile	1.6–1000	y = 1.4538x + 0.0510	0.9996	0.39
urine	1.6–1000	y = 0.8509x + 0.0192	0.9997	0.16
feces	0.64–400	y = 2.4378x + 0.0427	0.9996	0.45

**Table 2:** The PK characteristics of *α*-cyperone in rat plasma (*n* = 6, mean ± SD).

PK Characteristic	Intravenous Administration	Oral Administration
T_max_ (h)	0.03 ± 0.00	0.20 ± 0.16
C_max_ (ng/mL)	1258.68 ± 156.85	51.19 ± 16.41
AUC_(0→t)_ (μg/L × h)	380.62 ± 50.73	25.89 ± 14.01
T_1/2_ (h)	0.23 ± 0.17	0.14 ± 0.05
MRT_(0→t)_ (h)	0.58 ± 0.12	0.47 ± 0.22
MRT_(0→∞)_ (h)	0.58 ± 0.12	0.47 ± 0.22
F (%)	–	1.36

**Table 3:** The excretion characteristics of α-cyperone in rat bile, urine, and feces (*n* = 6, mean ± SD).

Sample	T_1/2_ (h)	Ke (h^−1^)
bile	2.10 ± 0.72	0.37 ± 0.13
urine	21.58 ± 24.81	0.06 ± 0.03
feces	18.84 ± 9.15	0.05 ± 0.04

**Table 4:** The ion transition parameters of α-cyperone and alantolactone in LC-MS/MS analysis.

Compound	Precursor Ion (*m*/*z*)	Fragment Ion (*m*/*z*)	Fragmentor Voltage (V)	Collision Energy (eV)
α-cyperone	219.0	111.0	134	25
alantolactone	233.1	151.0	110	9

## Data Availability

The data presented in this study are available upon request from the corresponding author.

## Supplementary Materials

The following supporting information can be downloaded at: https://www.mdpi.com/article/10.3390/molecules30193899/s1, Table S1. The intra- and inter-day precision and accuracy, extraction recovery, and matrix effect of α-cyperone in plasma, bile, urine, and feces; Table S2. The stability of α-cyperone in plasma, bile, urine, and feces; Table S3. The comparison of the established method with reported methods; Table S4. The process of sample collection from plasma, bile, urine, and feces.

## Abbreviations

The following abbreviations are used in this manuscript:

CR	*Cyperi Rhizoma*
UHPLC-QQQ-MS/MS	Ultra-performance liquid chromatography tandem triple quadrupole mass spectrometry
MRM	Multiple reaction monitoring
QC	Quality control
LLOQ	Lower limit of quantification
C_max_	Maximum plasma concentration
T_max_	Time to reach C_max_
T_1/2_	Half-life time
AUC	Area under the curve
F	Absolute bioavailability

## References

[1] Taheri Y., Herrera-Bravo J., Huala L., Salazar L.A., Sharifi-Rad J., Akram M., Shahzad K., Melgar-Lalanne G., Baghalpour N., Tamimi K. (2021). *Cyperus* spp.: A review on phytochemical composition, biological activity, and health-promoting effects. Oxidative. Med. Cell. Longev..

[2] Pan S., Zhang Z., Feng Q., Zhao J., Aziz S., Dou L., Li J., Huang L., Yan H., Wang X. (2023). An efficient high-speed counter-current chromatography method for the preparative separation of sesquiterpenoids from the rhizomes of *Cyperus rotundus* L. combined with evaluation of the anti-inflammation activity in vitro and molecular docking. J. Sep. Sci..

[3] Shi Y., Mei X., Li Y., Li M., Ji D., Su L., Mao C., Lu T. (2023). Study on the quality difference of *Cyperus rotundus* before and after vinegar processing based on ultra-high-performance liquid chromatography-quadrupole-time of flight-mass spectrometry and molecular network combined with color parameters. J. Sep. Sci..

[4] Wang F., Zhang S., Zhang J., Yuan F. (2022). Systematic review of ethnomedicine, phytochemistry, and pharmacology of *Cyperi Rhizoma*. Front. Pharmacol..

[5] Huang B., Hu G., Zong X., Yang S., He D., Gao X., Liu D. (2023). α-Cyperone protects dopaminergic neurons and inhibits neuroinflammation in LPS-induced Parkinson’s disease rat model via activating Nrf2/HO-1 and suppressing NF-κB signaling pathway. Int. Immunopharmacol..

[6] Zhang H., Li S., Lu J., Jin J., Zhu G., Wang L., Yan Y., He L., Wang B., Wang X. (2021). α-Cyperone (CYP) down-regulates NF-κB and MAPKs signaling, attenuating inflammation and extracellular matrix degradation in chondrocytes, to ameliorate osteoarthritis in mice. Aging.

[7] Daude R.B., Bhadane R., Shah J.S. (2024). Alpha-cyperone mitigates renal ischemic injury via modulation of HDAC-2 expression in diabetes: Insights from molecular dynamics simulations and experimental evaluation. Eur. J. Pharmacol..

[8] Ge N., Yan G., Sun H., Yang L., Kong L., Sun Y., Han Y., Zhao Q., Kang S., Wang X. (2023). Version updates of strategies for drug discovery based on effective constituents of traditional Chinese medicine. Acupunct. Herb. Med..

[9] Adolat M., Andrey B., Timur K., Igor Y., Pavel A., Madina A., Vladimir B., Vyacheslav D. (2025). Gallic, aconitic, and crocetin acids as potential TNF modulators: An integrated study combining molecular docking, dynamics simulations, ADMET profiling, and gene expression analysis. Molecules.

[10] Zhang C.X., Arnold S.L.M. (2025). Potential and challenges in application of physiologically based pharmacokinetic modeling in predicting diarrheal disease impact on oral drug pharmacokinetics. Drug. Metab. Dispos..

[11] Xi J., Qian D., Duan J., Liu P., Zhu Z., Guo J., Zhang Y., Pan Y. (2015). Preparation, characterization and pharmacokinetic study of Xiangfu Siwu Decoction essential oil/β-cyclodextrin inclusion complex. Molecules..

[12] Chuanhua F., Huiling G., Xiaolin T., Xiaojuan Z., Xinlu F., Dekun L., Gang L. (2023). Determination of cyperenone and α-cyperone in rat plasma by UPLC-MS/MS and their pharmacokinetics. Chin. J. Mod. Appl. Pharm..

[13] Nan G., Fanna M. (2009). Pharmacokinetics study of alpha-cyperone in rat. Chin. Med. Biotechnol..

[14] Bansal S., Paine M.F., Unadkat J.D. (2025). Predicting in vivo cannabinoid-drug interactions mediated via inhibition of UDP-glucuronosyltransferases using in vitro studies and physiologically based pharmacokinetic modeling and simulations. Drug. Metab. Dispos..

[15] Xuejiao W., Fei W., Peng T., Huiming H., Zhuguo W., Jinxin X., Longyan W., Dongxiao L., Zhongdong H. (2024). The interactions between traditional Chinese medicine and gut microbiota in cancers: Current status and future perspectives. Pharmacol. Res..

[16] Mödinger Y., Knaub K., Dharsono T., Wacker R., Meyrat R., Land M.H., Petraglia A.L., Schön C. (2022). Enhanced oral bioavailability of β-caryophyllene in healthy subjects using the VESIsorb(^®^) formulation technology, a novel self-emulsifying drug delivery system (SEDDS). Molecules.

[17] Ansari M.T., Batty K.T., Iqbal I., Sunderland V.B. (2011). Improving the solubility and bioavailability of dihydroartemisinin by solid dispersions and inclusion complexes. Arch. Pharmacal. Res..

[18] Chaves J.S., Leal P.C., Pianowisky L., Calixto J.B. (2008). Pharmacokinetics and tissue distribution of the sesquiterpene alpha-humulene in mice. Planta Medica.

[19] Zhai B., Zeng Y., Zeng Z., Zhang N., Li C., Zeng Y., You Y., Wang S., Chen X., Sui X. (2018). Drug delivery systems for elemene, its main active ingredient β-elemene, and its derivatives in cancer therapy. Int. J. Nanomed..

[20] Anand P., Kunnumakkara A.B., Newman R.A., Aggarwal B.B. (2007). Bioavailability of curcumin: Problems and promises. Mol. Pharm..

[21] Alizadeh S.R., Savadkouhi N., Ebrahimzadeh M.A. (2023). Drug design strategies that aim to improve the low solubility and poor bioavailability conundrum in quercetin derivatives. Expert. Opin. Drug. Discov..

[22] Wei C., Jiansong M., Chun L., Jinlian K. (2025). Alpha-cyperone ameliorates renal fibrosis and inflammation in mice with chronic kidney disease via NF-κB and Akt/Nrf2/HO-1 pathways. Immunopharmacol. Immunotoxicol..

[23] Huang B., Liu J., Fu S., Zhang Y., Li Y., He D., Ran X., Yan X., Du J., Meng T. (2020). α-Cyperone Attenuates H(2)O(2)-Induced Oxidative Stress and Apoptosis in SH-SY5Y Cells via Activation of Nrf2. Front. Pharmacol..

[24] Qiao N., Wang Q., Tao Y., Wu J., Fang Y., Ni Y., Ding X. (2023). α-Cyperone ameliorates depression in mammary gland hyperplasia and chronic unpredictable mild stress rat by regulating hormone, inflammation, and oxidative stress. Immunopharmacol. Immunotoxicol..

[25] Gao P., Ding N., Lv J., Ramzan M.N., Wen Q. (2021). α-Cyperone inhibitory effects on tumor-derived DNA trigger microglia by STING pathway. J. Ethnopharmacol..

[26] Kim C., Kim S., Jung A.R., Jang J.H., Bae J., Choi W.I.I., Sung D. (2024). Nanoparticle encapsulation of the hexane fraction of Cyperus Rotundus extract for enhanced antioxidant and anti-Inflammatory activities in vitro. Int. J. Nanomed..

