# Supplementary material for: The Pharmacokinetics, Bioavailability, and Excretion Studies of α-Cyperone in Rats by UHPLC-QQQ-MS/MS
Source: Molecules. 2025 Sep 26;30(19):3899. doi: 10.3390/molecules30193899 (PMC12525850; doi:10.3390/molecules30193899)
Supplement: Supplementary file 1 [file molecules-30-03899-s001.zip › molecules-3867685-supplementary.pdf]

**Supplementary materials**

# The pharmacokinetics, bioavailability and excretion studies of *α*-cyperone in rat by UHPLC-QQQ-MS/MS

Ye Shang [†], Yameng Zhu [†], Kaili Zhang [†], Zijing Zhang, Huining Geng, Xueyu Liu, Wenwen Li, Lu Chen, Caixia Li, Yang Liu, Huizi Ouyang, Jun He *

State Key Laboratory of Chinese Medicine Modernization, Tianjin University of Traditional Chinese Medicine, Tianjin, 301617, China.

*Corresponding author

E-mail: hejun673@163.com (J. H.)

Tel.: +86-22-59596163; Fax: +86-22-59596163

† Contributed equally to this work

**Table legend**

**Table S1**. The intra- and inter-day precision and accuracy, extraction recovery, and matrix effect of $\alpha$-cyperone in plasma, bile, urine, and feces.

**Table S2**. The stability of $\alpha$-cyperone in plasma, bile, urine, and feces.

**Table S3.** The comparation of established method with reported methods.

**Table S4.** The process of sample collection from plasma, bile, urine, and feces.

Table S1. The intra- and inter-day precision and accuracy, extraction recovery and matrix effect of $\alpha$-cyperone in plasma, bile, urine and feces.

| **Sample** | **Theoretical concentration (ng/mL)** | **Intra-day** | | **Inter-day** | | **Extraction recovery (%)** | **Matrix effect (%)** |
|---|---|---|---|---|---|---|---|
| | | **Precision (%)** | **Accuracy (%)** | **Precision (%)** | **Accuracy (%)** | | |
| plasma | 0.4 | 4.6 | −2.6 | 5.6 | −6.2 | 97.9 ± 5.6 | 97.6 ± 3.7 |
| | 60 | 5.4 | −14.5 | 3.8 | −3.1 | 103.8 ± 0.1 | 90.0 ± 0.0 |
| | 1200 | 12.4 | −9.0 | 8.4 | 1.4 | 100.1 ± 0.1 | 86.3 ± 0.0 |
| bile | 1 | 3.7 | 1.7 | 4.4 | 2.9 | 101.7 ± 4.4 | 95.5 ± 9.0 |
| | 40 | 2.9 | 2.7 | 0.7 | −0.5 | 114.6 ± 13.5 | 99.0 ± 4.5 |
| | 800 | 0.4 | −8.5 | 4.6 | −3.2 | 99.9 ± 15 | 90.6 ± 10.8 |
| urine | 0.4 | 4.0 | −10.4 | 5.6 | −5.9 | 87.1 ± 1.1 | 98.8 ± 6.3 |
| | 40 | 2.3 | −7.8 | 2.2 | −2.9 | 85.4 ± 4.6 | 91.1 ± 4.1 |
| | 800 | 3.0 | 1.4 | 1.4 | −0.9 | 85.4 ± 5.3 | 91.3 ± 2.9 |
| feces | 1 | 4.1 | 4.1 | 4.1 | 3.2 | 101.6 ± 9.2 | 83.7 ± 8.3 |
| | 16 | 11.0 | −9.2 | 1.6 | 0.4 | 99.0 ± 6.7 | 72.9 ± 5.3 |
| | 320 | 7.7 | 0.0 | 3.4 | −0.5 | 94.8 ± 2.7 | 81.5 ± 5.4 |

Table S2. The stability of *α*-cyperone in plasma, bile, urine and feces.

| Sample | Theoretical concentration (ng/mL) | Room temperature for 4 h | | Auto−sampler for 12 h | | Freeze−thawing for 3 cycles | | −80 °C for 7 days | |
|---|---|---|---|---|---|---|---|---|---|
| | | RE (%) | RSD (%) | RE (%) | RSD (%) | RE (%) | RSD (%) | RE (%) | RSD (%) |
| plasma | 0.4 | 2.6 | 5.2 | 3.7 | 5.2 | 4.0 | 2.3 | −2.5 | 5.3 |
| | 60 | −5.2 | 3.9 | −4.0 | 10.3 | 6.9 | 3.9 | 2.4 | 7.3 |
| | 1200 | 3.6 | 4.7 | 2.6 | 14.0 | −9.7 | 9.8 | −2.1 | 4.6 |
| bile | 1 | 1.1 | 10.4 | 3.8 | 6.0 | 0.3 | 9.2 | −1.5 | 4.1 |
| | 40 | −4.9 | 3.8 | −3.7 | 2.7 | −6.7 | 0.6 | −6.6 | 0.3 |
| | 800 | −6.1 | 2.6 | 2.5 | 0.7 | 0.3 | 0.7 | 0.2 | 0.6 |
| urine | 0.4 | 1.4 | 4.8 | 1.2 | 4.6 | 3.5 | 3.5 | −5.5 | 3.0 |
| | 40 | −3.9 | 3.0 | −3.9 | 2.6 | −4.9 | 2.0 | −0.5 | 2.9 |
| | 800 | −3.3 | 1.0 | −2.5 | 1.5 | 4.0 | 1.1 | 1.6 | 2.9 |
| feces | 1 | −0.9 | 10.5 | 3.4 | 5.5 | 1.1 | 10.0 | −2.9 | 5.3 |
| | 16 | 2.8 | 4.1 | 0.2 | 3.2 | −5.2 | 6.8 | −0.3 | 6.4 |
| | 320 | −9.2 | 6.7 | −0.9 | 7.7 | 0.9 | 4.8 | −4.3 | 5.2 |

Note: extraction recovery and matrix effect were calculated by the peak area of analyte in QC sample (A), post-treatment spiked sample (B), and standard solution (C) at the same concentration. Extraction recovery (%) = A/B × 100%. Matrix effect (%) = B/C × 100%.

Table S3. The comparation of established method with reported methods.

| Type of sample | Amount of sample | Type of extraction solvent | Extraction method | Detection method | LLOQ (ng/mL or ng/mg) | Detection time (min) | Reference |
|---|---|---|---|---|---|---|---|
| Plasma | 200 μL | ethyl acetate | vortex for 2 min | UPLC-TQ-MS/MS | 8.2 | 9 | [11] |
| Plasma | 100 μL | methanol | vortex for 2 min | UPLC-TQ-MS/MS | 0.5 | 8 | [12] |
| Plasma | 100 μL | acetonitrile | vortex for 5 min | HPLC-UV | 4.6 | 15 | [13] |
| Plasma, urine, bile, feces | 100 μL/ 25 mg | ethyl acetate/ acetonitrile | vortex for 3 min | UHPLC-QQQ-MS/MS | 0.15–0.45 | 3 | this work |


11. Xi, J.; Qian, D.; Duan, J.; Liu, P.; Zhu, Z.; Guo, J.; Zhang, Y.; Pan, Y., Preparation, characterization and pharmacokinetic study of Xiangfu Siwu Decoction essential oil/β-cyclodextrin inclusion complex. Molecules (Basel, Switzerland) 2015, 20, (6), 10705-20.
12. Chuanhua, F.; Huiling, G.; Xiaolin, T.; Xiaojuan, Z.; Xinlu, F.; Dekun, L.; Gang, L., Determination of cyperenone and $\alpha$-cyperone in rat plasma by UPLC-MS/MS and their pharmacokinetics. Chinese journal of modern applied pharmacy 2023, 40, (23), 3197-3201.
13. Nan, G.; Fanna, M., Pharmacokinetics study of alpha-cyperone in rat. Chinese medicinal biotechnology 2009, 4, (04), 312-314.

Table S4. The process of sample collection from plasma, bile, urine and feces.

| Sample | Collection process |
|---|---|
| plasma | pre-dose and 0.03, 0.08, 0.17, 0.25, 0.5, 0.75, 1, 2, 4, 6, 8, 10, 12, and 24 h post-dose |
| bile | pre-dose and 0 – 1 h, 1 – 2 h, 2 – 4 h, 4 – 6 h, 6 – 8 h, 8 – 12 h, and 12 – 24 h post-dose |
| urine | pre-dose and 0 – 2 h, 2 – 4 h, 4 – 6 h, 6 – 8 h, 8 – 12 h, and 12 – 24 h post-dose |
| feces | pre-dose and 0 – 6 h, 6 – 12 h, and 12 – 24 h post-dose |
